# Dadzine: Zine making with young fathers as a participatory and DIY approach to research

**DOI:** 10.1177/27538702241263387

**Published:** 2024-07-21

**Authors:** Laura Way

**Affiliations:** School of Arts, Humanities and Social Sciences, 4920 University of Roehampton, London, UK

**Keywords:** Zines, creative research, participatory research, DIY, young fatherhood

## Abstract

This article consider how zine making might be understood as a participatory and DIY approach to research through a focus on zine making workshops with young fathers. Drawing upon Fletcher's conceptualisation of ‘zine ethos’, zines’ DIY ethic, their democratic and participatory ideal and their transformative potential will each be considered reflexively, highlighting some of the ways these might be enacted through zine making in the context of research but also potentially constrained. This article extends existing empirical work concerning zines in the context of research and offers some fresh reflections on their value as a participatory and DIY approach to research.

## Introduction

**‘**Zines […] are self-published, low-budget, non-profit print publications. Originally born out of fandom; particularly sci-fi, music, sport and cultural iconography, they have long served as a significant medium of communication in various subcultures and considered a mainstay of the “do-it-yourself” creative movement’. (French and Curd, 2022: 77)

Historically zine was short for ‘fanzine’, emerging as early as the 1930s among science fiction fans (for an archive of examples, refer to https://fanac.org/), and though definitions may vary, the above definition of zines does a particularly effective job of summarising some of their key aspects and historical context. As the quote suggests, there is a history of zine making and sharing within some particular subcultures. Fanzines (zines focused on fandom, e.g. of a particular band/music), for example, appear a key aspect of the British punk movement from the 1970s onwards (Grimes, 2016; Worley, 2015) and for riotgrrrl (1990s onwards) zines offered a DIY form of activism and feminism (Radway, 2016). Quintela and Guerra (2020) make a pertinent point though that our understanding of zines is hindered by an overfocus on the Anglo-Saxon world and recent scholarship has expanded understanding of zines in a greater variety of contexts (see, e.g. Anggawi, 2022; Guerra, 2023; Guerra and Quintela, 2020a, 2020b; Tong, 2020).

I begin this article by briefly introducing participatory research, before further considering zines as a participatory research method, highlighting some of the key literature concerning this. In this section, I will turn to two ways of conceptualising zines in the context of research, seeing them as ‘resource’ and/or ‘creation’ (Way, 2017), offering some empirical examples of the second approach, in particular. From here, I will introduce the research study with young fathers, the rationale for zine making with these participants, and how zine creation was approached in this context. The remainder of the article will reflect critically on zines’ capacity as a DIY and participatory approach to research with young fathers, framing this discussion around three, at times overlapping, aspects of zine ethos: zines’ DIY ethic; zines’ democratic and participatory ideal; and zines’ transformative potential (Fletcher, 2017). Embedded in this discussion will be reflections and take-away learning from zine-making with young fathers as a DIY and participatory approach to research.

## Creative and participatory research methods

My zine-making with young fathers took place within a qualitative longitudinal study titled Following Young Fathers Further (FYFF) (led by Professor Anna Tarrant, University of Lincoln, UKRI funded). ‘Young father’ within this study was defined as those who had had or were expecting their first child at the age of 25 or under. This was reflective of UK policy and it is recognised that this is conceptualisation of ‘young’ is culturally located and not universal. I will return to the details of the zine making in FYFF below and speak to the literature on zines as a research method next, but I wish to pause here for a moment to consider this overall participatory approach to research (and research with young fathers specifically) more broadly.

Cornwall and Jewkes (1995) propose that the location of power across the research process is the key difference between participatory and other research methodologies. Indeed, participatory approaches recognise research ‘participants’ more so as ‘co-researchers’. Care needs to be taken, though, in being reflexive when engaging in such methods, recognising how participatory methodologies are grounded in practices in majority-world countries (Lenette, 2022). Related to this, Seppälä et al. (2021) highlight how arts-based methods can contribute to the decolonising of participatory research, including through their amplification of marginalised or devalued knowledges and supporting the perspectives of communities in their place-based/cultural contexts.

The value of participatory methodology with diverse young fathers has yet to be fully unpacked in sociological discussions concerning theory, practice, and method. Nevertheless, beyond FYFF, there are notable examples of creative, participatory methods with young fathers in research, such as the use of photovoice (Sopack et al., 2015), participant-led time maps, relationship maps and self-portraits in interviews (Neale et al., 2015), and peer research (Braye and McDonnell, 2012). Young fathers experience marginalisation at both a broader societal level but also with regards to being under-researched in family and parenthood research (Davies and Hanna, 2020). With regards to research with young fathers, then, the need for greater participatory methods, given their capacity for democraticising the research process and empowering those involved (Alminde and Warming, 2020), is clear. Underpinned by a collaborative and participatory approach to research, FYFF employed what it referred to as a ‘constellation’ of creative and/or participatory qualitative methods with young fathers to produce a rich and in-depth picture of their lives and support needs (Tarrant et al., 2023). This included recurrent interviews, the co-creation of father-inclusive interventions, zine making workshops and photovoice with young fathers and their children (Ladlow and Tarrant, 2023).

## Zines in the context of research

Qualitative research is increasingly utilising creative and participatory methods. Such methods allow new and innovative ways to engage participants in research projects, approaching research as that which is done *with* participants rather than being done *to* them. Zines, when used in the context of research, can be understood as a creative method and, depending on how zines are used, they can also be seen as a participatory method, for example, one which disrupts where the power lies amongst researchers and participants (Cornwall and Jewkes, 1995). It is this difference I will discuss first in this section on zines in the context of research.

Creative methods, more broadly, are typically those which involve participants expressing themselves in non-verbal ways, they can involve researchers focusing on ‘found’ materials or the researcher facilitating more participatory approaches involving creation and production (Brooks et al., 2020). Such a distinction is similar to that which I have used elsewhere when considering zines as a creative and/or participatory research method, in which I distinguish between ‘zines as resource’ and ‘zines as creation’ (Way, 2017). ‘Zines as resource’ refers to when zines are treated as secondary data with pre-existing zines being used by the researcher and analysed (.g.e through a content analysis, textual analysis and so forth). This aligns itself with the ‘found’ material approach to creative methods noted above (Brooks et al., 2020). Red Chidgey's (2013) work concerning young women's zines and feminist memory uses zines in this way, for example, in which a textual analysis of the print zine ‘Reassesses Your Weapons’ was carried out.

‘Zines as creation’, on the other hand, treats zines as primary data more so than secondary with zines produced as a part of research (e.g. they did not exist prior to the research). Katharine Houpt et al.'s (2016) work which involved zine-making with older adults in nursing homes would be an example of such an approach. Whilst both the resource and creation approaches entail zines being understood as a creative method, this distinction should make clear the potential for zines to be used as a participatory method too if taking a ‘zines as creation’ approach (reflecting too the approaches identified by Brooks et al., 2020 above). Such an approach has increased in prominence as a methodological tool amongst social researchers, whilst remaining an area fruitful for development. It is this ‘zines as creation’ approach to zines which was adopted in our research with young fathers and therefore that which I focus upon in this article. Before introducing the research with young fathers in which zine making took place though, I will next offer some overview of this zines as creation approach in the literature. This is by no means an exhaustive consideration of all existing examples nor a systematic review of literature but serves to offer some key themes through select examples to contextualise this work.

### Zines as creation

A growing number of empirical examples exist where this zines as creation approach has been utilised. Often, it concerns zines being made as a part of an in-person or online ‘zine workshop’, facilitated by a researcher and/or experienced ‘zinester’. Research by Lupton and Watson (2021) and Gray et al. (2022), for example, entailed participants creating individual zines whilst in a shared space (with this shared space being an online one in Gray et al.'s). The organisers of the ‘Welfare Imaginaries’ workshop series also took this approach but, additionally, collated the zine pages together with text from themselves to produce a finished zine which was made available online (Welfare Imaginaries, 2022). Ptolomey's (2020) research concerning disabled young women's everyday lives and imagined futures involved her facilitating zine making workshops with her participants with these individually produced zines then used as a precursor to interviews with participants. The interviews therefore comprised some reflection on the zine the participant made as well as other questions pertaining to the research's empirical focus, thus highlighting how zines can also become an elicitation tool in their use within qualitative research.

Zines as a participatory approach to analysis with participants has also been considered. Valli (2021) speaks to ‘interview-based zine making’ during which participants work with transcribed texts from researcher-conducted interviews. The aim being that participants engage with the perspectives of the interviewees, exploring disagreements and connections with their own thoughts with a collectively produced zine made for further dissemination. Along similar lines, Lamond et al. (2023), as a part of their Reclaim the (Night) Life project, have explored co-creational zine workshops as a way of supporting participant analysis of data which was captured in an earlier qualitative survey by the researchers, alongside the zines becoming a vehicle through which the young women involved could articulate their own experiences.

As alluded to above, zine workshops can be a place for collaboration between participants and researcher(s). In the Covid Realities (2022) research, for example, which carried out remote fieldwork during the pandemic and subsequent lockdown, online zine making workshops with participants were utilised alongside other creative and participatory methods. Their workshops comprised participants not just creating individual zine pages but them coming together again online to collaboratively decide how to present and organise the final zine itself. Such an approach was also used by the ‘Researchers don’t cry’ (2022) project in which online zine making workshops were proceeded by a ‘sharing symposium’ for participants to share creations and reflect on the process. Such collaborative zines therefore go beyond the call out for submissions approach which has been utilised within the zine community (by which a call is put out for submissions which the organiser then collates and distributes), involving instead more of a ‘back and forth’ process between participants and participants and researcher(s), the exact nature of which can be shaped by whether the workshops are online or in-person. This demonstrates how ‘the act of creative making is not simply a medium to facilitate or communicate research findings: it is a research generation practice in itself” (Lupton and Watson, 2021: n.p).

Concerning the afterlives of zines more so, some research has considered not just what zines can ‘do’ for those creating them, but also for those engaging with them thereafter. Houpt et al.'s (2016) research which concerned the creation of zines with nursing home community members (in-person) found that these zines became a way for the participants to share their experiences and ideas with other nursing home members and staff. Furthermore, Houpt et al. (2016) took the collaboratively created zine to conferences and festivals to share these ideas with the public and professionals more widely, making the voices of the nursing home community members heard as well as offering alternative narratives regarding ageing. This relates to one of the zine ethos points which will be discussed further below concerning zines’ transformative potential (Fletcher, 2017). In a similar vein, zine workshops have been utilised recently in online spaces as a feminist response to COVID-19 (Gray et al., 2022). This evidenced how zines could be used within the neoliberal academy (which reduces students to human capital and focuses on productivity of staff), to force open creative, feminist spaces of activism (Gray et al., 2022). It is worth raising here the seeming contradiction of using zines/zine making in neoliberal institutions, such as university, and similar concerns have been noted in relation to punk and academia/education more broadly (Furness, 2012). Taking Torrez's (2012) point though, ‘punkademics must fight to preserve counter-hegemonic sites of education within the corporate university […] to reclaim spaces of learning’ (p. 133).

So far in this article, I’ve considered zines as a research tool or method, focusing on a ‘zines as creation’ approach and highlighting some examples where such an approach has been used. I will turn now to offer some context concerning the research with young fathers and the zine-making which took place before moving into discussion of this in the context of what has been referred to as a zine ethos (Fletcher, 2017).

## Zine making with young fathers

As highlighted earlier, zine making was one of a number of methods employed by FYFF to form a ‘constellation’ of creative and/or participatory qualitative methods. Zine-making, via a ‘zines as creation’ approach, within this study, offered an additional way to generate insights concerning young fathers’ everyday and familial lives, providing a vehicle too through which they could share their thoughts and experiences (through the open distribution of the zine).

Four zine workshops were conducted with fathers during the FYFF study, one in-person and three online, with zine making responding to the provided prompt of ‘What does being a young dad mean to you?’. The workshops were facilitated by myself as a member of the FYFF research team and built upon my previous experiences of facilitating zine making workshops with learners within Further Education (Way, 2017) and utilising participant-created zine pages in research with older punk women (Way, 2020, 2024a, 2024b). In the spirit of this ongoing cumulative learning and reflection, whilst this article predominantly focuses on zine-making experiences with young fathers, there will be some connections made to my other experiences with zines in the context of research interwoven in its discussion.

The first zine workshop with young fathers which took place was the in-person workshop which involved two fathers who have been working with the FYFF team in developing/delivering in-person training and workshops, as ‘experts by experience’, with local professionals. The rationale here was that not only could zine making generate additional insights to sit alongside the qualitative interviews they had engaged in but could potentially become something they themselves might use as a tool in their local work with professionals. Already being engaged in in-person work with FYFF meant their zine workshop followed suit. I took materials with me to the workshop which might be used for zine making (such as pens, paper, newspapers, scissors, and glue) and, at the end of the workshop, I took photos of the zine pages that the two dads had created. The dads said they wished for me to take the original physical copies away with me too.

Returning to the wider cohort of young dads who had been engaged in the FYFF study through sustained involvement in our waves of interviewing, the choice to run the further zine workshops online reflected their preference for remote fieldwork methods (they had chosen, e.g. to take part in telephone and online interviews rather than in-person interviews across their longitudinal involvement). These workshops were then advertised to the fathers we had been researching with more broadly in FYFF, promoted by our various project partners, but also advertised on social media beyond this to broaden the sample, with seven dads participating in total. An advert for participants was constructed, stipulating that participants would not be expected to use their web cameras or microphones during the workshop, hoping this might encourage participants who might otherwise be put off by a need to be visible and/or the perception they would have to speak. The advert made clear that the workshop was free, that no previous knowledge of zines was required, that they would receive a £20 voucher to thank them for being involved, and that all they needed was a pen and an A4 piece of paper. Regarding the latter, after being introduced to zines and shown some examples, participants were then invited to grab any further materials at their disposal which they may wish to use. The fathers who attended the online workshops were asked if they could take a photo of their finished zine page(s) and e-mail these to the researcher. All bar one participant did.

The photographed zines pages were collated and uploaded to an online platform called Issuu, which allowed for them to be constructed and presented as an electronic zine, mirroring the turning of pages and layout of a physical zine. This electronic format makes sharing particularly easy though there are also plans for making physical copies of the zine in the future. This also meant further zine pages could be added, as and when workshops took place, resulting in a zine which is in some respects never finished, but always in process. The ‘dadzine’ (in its current, ‘ongoing’, form) can be viewed online on the FYFF website.

This article predominantly draws upon a reflexive analysis of my fieldwork notes (taken before and after the zine workshops). Additionally, there is some consideration of interview data from the young fathers involved in FYFF – the longitudinal approach taken in our research with young fathers meant we were able to capture their reflections on the zine making in our next wave of interviewing (all bar one of the young fathers who participated in the zine making were ongoing participants in the FYFF study more broadly). This focus on the *process* of zine making/writing responds to Quintela and Guerra's (2020) point that our understanding of zines previously has been limited due to the lack of relevance of the writing process.

The purpose of this article is not to present a detailed analysis of the zine pages produced (indeed, we are not yet at the stage of detailed analysis) but a brief comment is offered here, in part to lay some groundwork for some of the discussion concerning zine ethos below. Together, the zine pages reflected various themes which had been identified in interviews with young fathers by the FYFF team over the course of the longitudinal study. In particular, the narratives crafted within the zine pages confirmed a commitment to engaged fatherhood, or ‘being there’ for their children (Andreasson et al., 2023; Neale and Davies, 2015). Just as was found across the FYFF study, this commitment could be facilitated but also constrained by various factors, including employment and support. The zine pages the young fathers created, whilst highlighting the ‘rewards’ of fatherhood, highlighted, too, the challenges.

Earlier in this article when defining zines, I referred to how they can also be considered as comprising a particular ethos. In the next part of this article, I return to this and unpick it further, reflecting on my zine making with young fathers in considering zines as a DIY and participatory method.

## Zine making as a DIY and participatory method with young fathers

In reflecting on zine making as a DIY and participatory method, it is useful here to consider further this notion that zines comprise a particular ethos and, in doing so, I draw here upon Natalie Fletcher's (2017) paper in structuring this discussion. Fletcher highlights five key defining features relevant to zines’ ethos: their democratic and participatory ideal; their do-it-yourself ethic; their experimental spirit; their politicising influence; and their transformative potential. I will focus on three of these features in particular here which were felt to speak most to the zine making with young fathers—zines’ DIY ethic, zines’ democratic and participatory ideal, and zines’ transformative potential.

### Zines’ DIY ethic

As noted earlier, zines are associated with, or seen as an aspect of, DIY culture(s) and the making of and participation in zines, then, can be understood as embodying a DIY ethic (Fletcher, 2017) or ethos (Watson and Bennett, 2021). Zines’ association with ‘DIY’ can be understood more so when considered in tandem with punk and as Quintela and Guerra (2020) note, it was with punk that (fan)zines became a ‘materialisation’ of the DIY ethic/ethos. DIY is a self-conscious political act, concerned with the active creation of alternative culture as opposed to just passively consuming existing dominant culture (Duncombe, 1997). The making of zines embodies this DIY ethic, promoting the belief that ‘anyone can do it’ and to embrace amateurism (Fletcher, 2017).

When facilitating zine workshops, I always spend some time at the beginning exploring with participants what zines are and what might constitute some key elements of them, bringing some of my own zine collection along to help with this exploration. This groundwork is particularly important when workshops involve participants with no previous awareness and/or knowledge of zines, as has been the case with young fathers (only two had some familiarity with zines). This is also important when considering Duncombe's (1997) point that DIY is a *self-conscious* political act and the extent then this aspect of zines is realised in the context of facilitated (and prompted) zine making. Without doing this groundwork and exploring what zines can be, why they might be created and how they might be used, it is questionable whether this self-consciousness would be enacted.

Some link DIY to anarchist ideas concerning cooperating with others too to enact change (O’Hara, 1999). This presents DIY, then, as comprising of a ‘collective independence’ (Martin-Iverson, 2014: 187), valuing autonomy as well as community. It is important then to also consider zines as a collective practice. This might be clearly understood when considering people coming together to create collaborative zines, for example, but there are also the less tangible aspects such as those who participate in zine culture (whether through reading, collecting and/or making) feeling part of a wider zine community and these actions taken together presenting a collective voice and action against dominant culture. When facilitating zine workshops with young fathers, knowing they were contributing towards a collaborative zine indeed helped capture this collective practice. Community building could emerge through the sharing of the zine and the zine being engaged with by others (though there is currently no empirical basis for claiming the extent to which this has happened in our research with young fathers). In one of the online zine workshops, two fathers (unknown to each other before the workshop) did choose to keep their mics and cameras on and chatted whilst making their zine pages, making a new connection based upon their shared identity as fathers and talking about their experiences of fatherhood. The collaborative, collective aspect of zine making could have been extended by following the Covid Realities’ (2022) example of following up zine making workshops with further workshops in which participants were brought together to discuss how to collate and present the zine.

It is important to note that the DIY ethic, more broadly, has been criticised in its claim of ‘anyone can do it’ (Dale, 2008) and such a claim belies the inherent, close-minded privilege at its core (Phoenix, 2020). As Stewart and Way (2023, n.p) note ‘Saying anyone can do it, doesn’t make it so’ and they have highlighted the ableist privilege such a claim rests upon. Critiques have indeed been made concerning whose voices are raised in zine making and sharing (Nguyen, 2012). This is pertinent too when considering zines’ DIY ethic and the presentation of zine making and participation as accessible to all and it is also perhaps particularly important when considering contexts where the ‘doing’ has been facilitated by someone other than the zine maker(s). Fletcher (2017) notes, for example, in situations where zine making is introduced by an adult to youth, there is the question of whether adult authority (e.g. through guidance) can be a threat to the DIY ethic. This then questions the *active* creation involved in DIY culture such as zines (Duncombe, 1997) in such contexts where zine making has been prompted by a workshop facilitator, researcher and so forth. Here, in my workshops with young fathers, I felt it helped to remain as open as possible the focus of the zine pages and keep prompting to a minimal, as well as ensuring participants chose to take part in the zine workshop rather than being a ‘captive audience’ such as might be the case with a group of students led by a teacher in zine making (Way, 2017).

### Zines’ democratic and participatory ideal

Zines’ democratic and participatory ideal refers to how they aim for inclusion, to breakdown hierarchies between participants and value multiple voices (Fletcher, 2017). Related to this, French and Curd (2022) note how ‘Historically, zines have been an underground way for marginalized communities to record their stories, share information and organize’ (p. 78) and they have the capacity to provide visibility for those often marginalised in academic research (Gray et al., 2022; Ramdarshan Bold, 2017). In my research with older punk women, I understood zines as a way of providing such visibility to those who had been marginalised in academic research and this rationale followed too when researching with young fathers. Young fatherhood has not only been marginalised in academic research concerning fatherhood, families and parenthood more broadly (Lau Clayton, 2016) but evidence confirms that young fathers experience marginalisation in society, government policy, and in professional contexts (Tarrant et al., 2023b). Creating and dissemination a zine by young fathers, *on* young fatherhood, could go some way then in raising visibility of their thoughts and experiences.

Zines’ participatory ideal indicates, perhaps unsurprisingly, that zines are well suited as a method to be utilised if engaging in research which seeks to be grounded in a participatory ethos such as was the case with FYFF. As Ptolomey (2020) notes, zines can be a medium for agency and this sits particularly well too with such an ethos and can be a means of democratising the research process (Edwards and Brannelly, 2017). Returning to zines’ democratic ideal, their capacity to be ‘multimodal’ (Ptolomey, 2020) arguably opens up opportunities for a range of participants to engage in zine making. They do not, for example, necessitate artistic ability nor do they rely on literacy. This can make zine making an adaptable and accessible methodological tool (though this notion of being ‘open to all’, as demonstrated earlier, might be problematised).

Previously, when I utilised zine making in my research with older punk women, their zine pages were created individually and in their own time, for example, no workshops were involved. Reflecting on this afterwards, I noted a relative freedom in aesthetics and approach across their pages and wondered whether being in a zine workshop with provided materials might have constrained this (Way, 2024a). Interestingly, the zine pages produced in the in-person zine workshop with young fathers, in which materials were provided, did not make use of any ‘cut and stick’ materials with pages comprising of handwritten text/drawings and this was the case too, bar one, with those produced in the online zine workshops. A contributing element here too might have been time. My approach with older punk women entailed them having a fairly extended length of time to work on their zine pages (in most cases, a couple of months). This time could mean participants being able to think more so about what they wanted to produce, explore different approaches and potentially ‘source’ things for their zines (Way, 2024a), something more in keeping perhaps with how zinesters approach zine making. Again, this could have factored into the approach taken by the fathers given that the workshops were only 2 hours. I did make clear to the participants they could take their zine page(s) ‘away’ from the workshop itself to continue with/add to, if they wished. Only one young dad took up this offer on the basis and interestingly his zine pages did move away from the handwritten text/drawing approach the others had taken.

### Zines’ transformative potential

Fletcher (2017) argues that because of the other aspects of zines’ ethos (e.g. being grounded in a DIY ethic, possessing a participatory and democratising ideal…), they have the potential to be transformative for those engaged in them. As noted above, zines’ ability to provide some visibility for previously marginalised voices formed a key part of the rationale for their use with young fathers. Zine making workshops facilitated an opportunity for these fathers to challenge the prevailing deficit and stigmatising views of them found in society (Tarrant et al., 2023c), for example government policy, academic research and professional practice. Zines therefore were being understood as capable of being transformative not just for those engaged in making them, but in terms of those engaging with them after their creation when shared.

Being involved in participatory research can be empowering (Titterton and Smart, 2008) and Houpt et al.'s (2016) research, described earlier in this article, noted personal benefits of zine making for those involved. Reflecting on my use of zine making with older punk women, this was something I was unable to retrospectively gauge (Way, 2024a) and I had not built any ways into that research for the women to feedback or reflect upon their zine pages and making. However, as noted earlier, aided by the longitudinal approach taken in our research with young fathers, this was something we could capture their reflections on. All bar one of the young fathers who participated in the zine-making were ongoing participants in the FYFF study more broadly, and this facilitated the team being able to ask them about their zine making experiences in a subsequent wave of interviewing. Responses were positive and the fathers highlighted how zine making offered new modes for them to share their thoughts and experiences, through ways they had not previously (e.g. drawing or prose). Some of the dads also saw a value in zines as a method for sharing insights and experiences with their peers as well as with professionals/practitioners. This quote from our participant, Simon, is illustrative of some of these views:

It [the zine making] was really good. I think like the information was shared out in a different way and a bit of, it targets people of all ages and obviously there's so many different ways of getting information out there or sharing a story or a…moment in life or just like getting people's expressions out there in different ways […] And it's just, it's just so easy to get information over like a, a share of an experience or someone's opinion and it not feel really informal but it, it is, like it's hard to explain.

There are connections to be made between this transformative potential of zines and ethical research guidance and, arguably, the latter has the potential to constrain or limit the former. As with any approach to research, visual research methods, such as zines, generate ethical challenges. Lomax (2020) notes particularly how visual research methods often come into conflict with formalised ethical frameworks or guidelines, for example, concerning principles of anonymity and confidentiality. Zine pages can be anonymised—indeed, guidance can be provided in zine making workshops with participants to ensure this, for example, not featuring name(s), not including personal identifying details or images, with further anonymisation carried out by the researcher if required prior to the zine being shared/disseminated. This firstly raises the issue, however, of limiting the zines’ participatory and democratic ideal, by constraining participants’ autonomy in deciding what/how to share. Secondly, this has the potential of limiting the zines’ transformative potential if participants are stopped from owning their authorship. Related to this, remaining unnamed can undermine participant labour (Downes et al., 2013). In the FYFF study, the ‘afterlife’ of the zine was discussed with participants to help them make their own decisions concerning anonymity—none of them wished to be anonymised, reflecting how young fathers will engage in research because they want to make a difference and affect change (Ladlow and Tarrant, 2023). This highlights the importance for ethical consideration to be situated and context driven (Kousholt and Juhl, 2023; Lenette et al., 2018), as well as recognising ways ethical frameworks can limit some of the ethos of zines which might be key to our rationale for using them in research in the first place.

Moving beyond personal transformation to thinking about transformation more broadly, it would be difficult to gauge to what extent change is enacted by the sharing of the zine though facilitating an opportunity for making these participants’ voices heard is a step in the right direction for change (Houpt et al., 2016). Related to this though, zines, in our research with young fathers, contributed to our broader promotion and advocacy of father-inclusion through mechanisms of participation, co-production and investing in the capabilities/strengths of young fathers (Tarrant, 2023; Tarrant et al., 2023c), under a social engagement framework (Neale and Tarrant, 2024). Zines therefore offered another mode to facilitate the social participation of young fathers through the making of their contributions and these then being shared as part of a zine beyond the participants themselves.

## The final page

Despite their association with DIY cultures and/or their DIY ethic, this article has illustrated how this DIY component of zines cannot be assumed as an inevitability when utilising zines in the context of research. Indeed, there can be aspects of the research process and the research context which can constrain or limit such aspects, not least some of the associated ethical and practical considerations involved in doing creative and/or participatory research. Reflecting critically on the zine making with young fathers, however, also highlights the value of such a method, particularly in terms of zines’ democratic and participatory ideal, and their transformative potential. Zine making as part of a ‘constellation’ of creative and participatory methods with young fathers within the FYFF study has enabled our facilitation of their inclusion in knowledge creation, provided an opportunity for them to offer counter discourses to the common deficit approach to understanding young fatherhood, and offered a mode for their social participation under a social engagement framework.
